# Hypothalamic GPR40 Signaling Activated by Free Long Chain Fatty Acids Suppresses CFA-Induced Inflammatory Chronic Pain

**DOI:** 10.1371/journal.pone.0081563

**Published:** 2013-12-12

**Authors:** Kazuo Nakamoto, Takashi Nishinaka, Naoya Sato, Mitsumasa Mankura, Yutaka Koyama, Fumiyo Kasuya, Shogo Tokuyama

**Affiliations:** 1 Department of Clinical Pharmacy, School of Pharmaceutical Sciences, Kobe Gakuin University, Kobe, Japan; 2 Bizen Kasei Chemical Co. Ltd., Okayama, Japan; 3 Laboratory of Pharmacology, Faculty of Pharmacy, Osaka Ohtani University, Osaka, Japan; 4 Biochemical Toxicology Laboratory, Faculty of Pharmaceutical Sciences, Kobe Gakuin University, Kobe, Japan; University of Arizona, United States of America

## Abstract

GPR40 has been reported to be activated by long-chain fatty acids, such as docosahexaenoic acid (DHA). However, reports studying functional role of GPR40 in the brain are lacking. The present study focused on the relationship between pain regulation and GPR40, investigating the functional roles of hypothalamic GPR40 during chronic pain caused using a complete Freund's adjuvant (CFA)-induced inflammatory chronic pain mouse model. GPR40 protein expression in the hypothalamus was transiently increased at day 7, but not at days 1, 3 and 14, after CFA injection. GPR40 was co-localized with NeuN, a neuron marker, but not with glial fibrillary acidic protein (GFAP), an astrocyte marker. At day 1 after CFA injection, GFAP protein expression was markedly increased in the hypothalamus. These increases were significantly inhibited by the intracerebroventricular injection of flavopiridol (15 nmol), a cyclin-dependent kinase inhibitor, depending on the decreases in both the increment of GPR40 protein expression and the induction of mechanical allodynia and thermal hyperalgesia at day 7 after CFA injection. Furthermore, the level of DHA in the hypothalamus tissue was significantly increased in a flavopiridol reversible manner at day 1, but not at day 7, after CFA injection. The intracerebroventricular injection of DHA (50 µg) and GW9508 (1.0 µg), a GPR40-selective agonist, significantly reduced mechanical allodynia and thermal hyperalgesia at day 7, but not at day 1, after CFA injection. These effects were inhibited by intracerebroventricular pretreatment with GW1100 (10 µg), a GPR40 antagonist. The protein expression of GPR40 was colocalized with that of β-endorphin and proopiomelanocortin, and a single intracerebroventricular injection of GW9508 (1.0 µg) significantly increased the number of neurons double-stained for c-Fos and proopiomelanocortin in the arcuate nucleus of the hypothalamus. Our findings suggest that hypothalamic GPR40 activated by free long chain fatty acids might have an important role in this pain control system.

## Introduction

Inflammatory chronic pain, such as arthritis pain, joint pain and inflammatory bowel disease, is a significant health problem, and is initiated by tissue damage or inflammation [1]. At present, such pain is generally treated with antidepressants, anticonvulsants and cyclooxygenase inhibitors, but negative side effects remain [2]. As the molecular and cellular basis for the development and persistence of pain after inflammation remain unknown, it is difficult to understand the mechanisms underlying inflammatory pain and to develop new therapeutics.

Recently, endogenous *n*-3 series polyunsaturated fatty acids (PUFAs) or their derived lipid mediators have been found to have crucial roles in the local control and programming of acute inflammatory response and its resolution [3]. Both basic and clinical studies have shown that a dietary intake of *n*-3 PUFAs results in a reduction of pain associated with rheumatoid and inflammatory joint pain [4], [5], dysmenorrheal pain [6], fibromyalgia [7] and neuropathy [7]. On the other hand, there are several reports that resolvins D1 and E1, derived from docosahexaenoic acid (DHA) and eicosapentaenoic acid (EPA), respectively, could effectively reduce inflammatory [8] and postoperative pain [9].

Accumulating evidence clearly shows that free fatty acids (FFAs) can act as ligands for several G-protein-coupled receptors (GPCR), including GPR41 [10], GPR43 [10], GPR84 [11], GPR40 [12] and GPR120 [13]. Of these receptors, GPR40 is activated by long-chain fatty acids, such as DHA and EPA [14]. The activation of GPR40 triggers the phospholipase C (PLC)/1,4,5-triphosphate formation (IP_3_) signaling pathway and leads to intracellular Ca^2+^ mobilization. GPR40 activation in pancreatic β-cells causes insulin secretion in response to increased blood glucose levels. In the central nervous system (CNS), GPR40 is predominantly expressed in the subventricular zone as well as in newborn and mature neurons [15]. Although there are several reports that GPR40 signaling in the brain may contribute to generating new neurons for learning and memory [15], [16], [17], few studies have examined the role of GPR40 under physiological conditions.

It has been reported that DHA produces an antinociceptive effect via β-endorphin release in response to various pain stimuli similar to those used in the present study [18], [19]. Furthermore, we have demonstrated that DHA-induced antinociception via β-endorphin release might be mediated (at least in part) through GPR40 signaling in the supraspinal region [20]. Therefore, we have proposed that brain GPR40-mediated systems acting upon DHA could be novel pain-regulating molecules. While there is growing interest in targeting long-chain fatty acid-sensing GPR40 for its involvement in pain relief [21], few studies have examined the DHA-GPR40 signaling pathway in inflammatory pain.

In the present study, these issues were addressed using a complete Freund's adjuvant (CFA)-induced inflammatory model in mice, a well-characterized model of inflammatory pain. The functional role of hypothalamic GPR40 during inflammatory chronic pain was examined, and the involvement of astrocytes in the mechanisms of GPR40-induced antinociception was estimated.

## Materials and Methods

### Animals and Ethics Statement

The present study was conducted in accordance with the Guiding Principles for the Care and Use of Laboratory Animals adopted by the Japanese Pharmacological Society. All experiments were approved by the Ethical Committee for Animal Experimentation of Kobe Gakuin University (approval number A13–23; Kobe, Japan). Male ddY mice (age, 4 weeks) were obtained from Japan SLC (Hamamatsu, Japan). Mice were housed in cages at 23–24°C with a 12-h light–dark cycle (lights from 8 am to 8 pm) and food and water *ad libitum*.

### Drugs and Administration schedule

DHA (50 µg/mouse; Ikeda Tohka Industries Co., Ltd., Fukuyama, Japan), the selective GPR40-agonist GW9508 (1.0–25 µg/mouse; Cayman Chemical Co., Ann Arbor, MI, USA) and the GPR40 antagonist GW1100 (1.0–10 µg/mouse; Cayman Chemical Co.) were dissolved in 1% dimethyl sulfoxide (DMSO; Sigma-Aldrich Japan K.K., Ishikari, Japan) and the solution was diluted with saline before von Frey testing (1% DMSO final concentration). The doses of GW9508 were chosen based upon our previous publication [20], whereas GW1100 was selected on the basis of previous reports [22] and our preliminary experiments. Under a non-anesthetized state, DHA and GW9508 were administered via the intracerebroventricular (i.c.v.) route 10 min before CFA injection, and GW1100 was administered via the i.c.v. route 10 min before GW9508 injection. Flavopiridol (5 and 15 nmol/mouse; Santa Cruz Biotechnology, Inc., Santa Cruz, CA, USA), a cyclin-dependent kinase inhibitor, was administered by i.c.v. injection into the left lateral ventricle of the mice twice a day (at 9:00 and 19:00) after CFA treatment.

### The i.c.v. injection

The i.c.v. injection was performed by using a Hamilton microsyringe fitted with a 27-gauge i.c.v. needle [23]. The injected site was both 2 mm caudal and lateral to the bregma and 3 mm in depth from the skull surface. Injection volumes were 5 µL introduced over 5 s. Verification of needle position in the lateral cerebroventricle was made by i.c.v. dye injection and subsequent *post-mortem* confirmation of dye placement within brain sections.

### Complete Freund's Adjuvant-induced inflammatory chronic pain mouse model

This protocol was conducted as previously described [8]. Briefly, persistent inflammatory pain was produced by intraplantar (i.pl.) injection of CFA (10 µL, 0.5 mg/ml; Sigma-Aldrich Japan K.K.) into the plantar surface of a mouse hind paw.

### Hyperplasia

Hyperplasia of paw tissue was measured by means of a digital caliper (Shinwa Co., Ltd., Niigata, Japan) before and at several times after i.pl. CFA injection during the 1- or 7-day period of study.

### Mechanical allodynia

Mechanical allodynia was evaluated using von Frey filaments (Neuroscience Inc., Tokyo, Japan) as previously described [24]. Mice were placed on a 5×5 mm wire mesh grid floor, covered with an opaque cup to avoid visual stimulation, and allowed to adapt for 2–3 h prior to testing. The von Frey filament was then applied to the middle of the planter surface of the hind paw with a weight of 0.16 g. On the indicated days, withdrawal responses following hind paw stimulation were measured 10 times, and mechanical allodynia was defined as an increase in the number of withdrawal responses to the stimulation. To test the effect of GW9508 or DHA on mechanical allodynia at 1 or 7 days after CFA injection, the von Frey test was performed on the mice at 10, 20, 30 and 60 min after DHA or GW9508 i.c.v. injection. Flavopiridol-treated mice underwent the von Frey test after 1 or 7 days after CFA injection.

### Thermal hyperalgesia

Thermal hyperalgesia of the hind paw was assessed using the plantar test (Ugo Basile Srl, Comerio VA, Italy), according to a previously described methodology [25]. Briefly, mice were acclimatized to an apparatus consisting of individual Perspex boxes on an elevated glass table, and an infrared radiant heat (40 W) source was directed onto the plantar surface of the hind paw, with the withdrawal response defined as the paw withdrawal latency. The heat application cut-off point was set at 20 s to prevent tissue damage. The apparatus was calibrated to give a paw withdrawal latency of ∼10 s in intact mice. To test the effect of GW9508 or DHA on thermal hyperalgesia at 1 or 7 days after CFA injection, the plantar test was performed on the mice at 30 min after DHA or GW9508 i.c.v. injection. Flavopiridol-treated mice underwent the plantar test after 1 or 7 days after CFA injection.

### Sample preparation for FFAs in the hypothalamic tissue

The wet weight of hypothalamic tissue was measured, and that tissue was homogenized in methanol/acetone (1∶1 v/v, containing 1 mg/ml of C19:0 tuberculostearic acid as internal standard solution including C19:0 tuberculostearic acid). The homogenate was centrifuged at 16,000 g for 5 min at 4°C. The resulting supernatant was moved into a glassy vial as the analysis sample of each FFA. To measure C12:0, C14:0, C15:0, C16:0, C16:1, C17:0, C18:0, C18:1, C18:2, C18:3, C20:0, C20:3, C20:4, C20:5 and C22:6, the supernatant was diluted by a factor of 10 or 50 [26].

### FFAs comparative analysis

FFAs comparative analysis was measured as previously described with some modifications [26]. The composition of FFAs was analyzed with the UHPLC-MS/MS system (Ultra high performance liquid chromatography, Nexera; MS:LCMS-8030 triple quad 5500 mass spectrometry, Shimadzu Co., Kyoto, Japan) controlled by LabSolutions LCMS version 5.4 (Shimadzu Co.). To perform the relative concentration assessment, the peak area values obtained from the NMR chromatogram of each fatty acid were normalized using that of C19:0 tuberculostearic acid as an internal standard. Next, the amounts of each fatty acid in the hypothalamus extract, with and without CFA treatment, were calculated, subtracting the results of each negative control sample from those of the corresponding hypothalamus tissue extract. HPLC separation was performed on a Mightysil RP-18(L) GP column (2 mm I.D.×10 cm, 5 µm particle size). The mobile phases were gradients of 10 mM ammonium acetate/methanol. The flow rate was set to 0.3 mL/min.

### Western blot analyses

Western blotting was done as previously described [20] with some modifications. Hypothalamus tissue was homogenized in homogenization buffer. Protein samples (20 µg) were resolved by 15% sodium dodecyl sulfate-polyacrylamide gel electrophoresis and transferred onto nitrocellulose membranes (Bio-Rad Laboratories, Inc., Hercules, CA, USA). GPR40 (1∶500; Abcam, Inc., Cambridge, MA, USA) was then assessed using rabbit polyclonal primary antibodies, and glial fibrillary acidic protein (GFAP 1∶1000; Millipore Corp., Billerica, MA, USA) was detected using mouse monoclonal primary antibodies. Glyceraldehyde-3-phosphate dehydrogenase (GAPDH) was used as a loading control and was detected using primary antibodies (1∶20,000; Chemicon International Inc., Temecula, CA, USA). Blots for GPR40 and GFAP were incubated overnight with the primary antibody at 4°C in Tris-buffered saline containing 0.1% Tween-20 and blocking agent (GE Healthcare, Tokyo, Japan). After washing, blots were incubated with horseradish peroxidase (HRP)-conjugated anti-rabbit IgG for GPR40 (1∶1000; KPL, Inc. Gaithersburg, MD, USA) and HRP-conjugated anti-mouse IgG for GFAP and GAPDH (1∶1000 and 1∶10000, respectively; KPL, Guildford, UK) for 1 h at room temperature. Immunoreactive bands were visualized using a Light-Capture system (AE-6981; ATTO Corp., Tokyo, Japan) with an ECL™ Western Blotting Analysis System (GE Healthcare, Buckinghamshire, UK). The signal intensities of immunoreactive bands were analyzed using a Cs-Analyzer (Ver. 3.0; ATTO Corp.).

### Brain tissue preparations

Mice were deeply anesthetized with sodium pentobarbital (65 mg/kg) and perfused transcardially with phosphate-buffered saline (PBS), pH 7.4, followed by 4% paraformaldehyde in 0.1 M PBS, pH 7.4. Brain sections were collected, post-fixed in 4% paraformaldehyde for 3 h, and dehydrated in 10% sucrose at 4°C for 3 h, and 20% sucrose at 4°C overnight. The following day, tissues were frozen in optimal cutting temperature compound (Tissue-Tek OCT Compound, Sakura Finetek Japan Co., Ltd. Tokyo, Japan) and held at −80°C until use. Sections were cut at 15 µm with a cryostat (CM1850, Leica, Microsystems GmbH, Wetzlar, Germany), and mounted on an MAS-coated glass slide (S9115, Matsunami Glass Ind., Ltd., Osaka, Japan).

### Double immunofluorescence study

The sections were washed with PBS containing 0.1% Triton X-100 (PBST) three times at 5 min intervals and incubated with blocking buffer (3% BSA in PBST) for 2 h at room temperature. The sections were then incubated in specific antibodies against GPR40 (rabbit polyclonal anti-GPR40, 1∶200, Abcam, Inc.,), or GPR40, which was prelabeled with the Zenon Alexa Fluor 594 Rabbit IgG Labeling Kit (Life Technologies, Carlsbad, CA, USA). NeuN (anti-mouse NeuN monoclonal antibody, 1∶1000; Millipore Corp.), GFAP (mouse monoclonal antibody anti-GFAP, 1∶1000; Millipore Corp.), proopiomelanocortin (POMC) (goat polyclonal anti-POMC; 1∶100; Abcam, Inc.), c-Fos (rabbit polyclonal anti-cFos antibody, 1∶2000; Santa Cruz Biotechnology) and β-endorphin (rabbit polychronal antibody anti-β-endorphin, 1∶100; Bioss Inc., Woburn, MA, USA) were stored overnight at 4°C, at which time the antibody was diluted in reaction buffer (0.01% Triton X-100 and 1% BSA in PBS). The next day, sections were washed with PBST and incubated in secondary antibody conjugated with AlexaFluor 488 and/or 594 (donkey polyclonal anti-rabbit IgG and goat polyclonal anti-mouse IgG 1∶200, Life Technologies, Inc.) at room temperature for 2 h, at which time the secondary antibody was diluted with reaction buffer. Finally, sections were washed with PBST and coverslipped with Perma Fluor (Thermo Shandon Inc., Pittsburgh, PA, USA), and immunoreactivity was detected with a confocal fluorescence microscope (FV1000, Olympus Corporation, Tokyo, Japan). In the immunohistochemical control studies, no staining was detected when the corresponding primary or secondary antibody was omitted.

### Statistical analyses

Data were expressed as mean ± S.E.M. Significance differences were evaluated by one-way analysis of variance followed by Dunnett's or Scheffe's multiple-comparison tests for comparisons between more than three groups or by Student's *t*-test for comparison between two groups. A *p* value of <0.05 was regarded as significant.

## Results

### Development of hyperplasia, mechanical allodynia and thermal hyperalgesia after CFA injection

**Figure 1:**
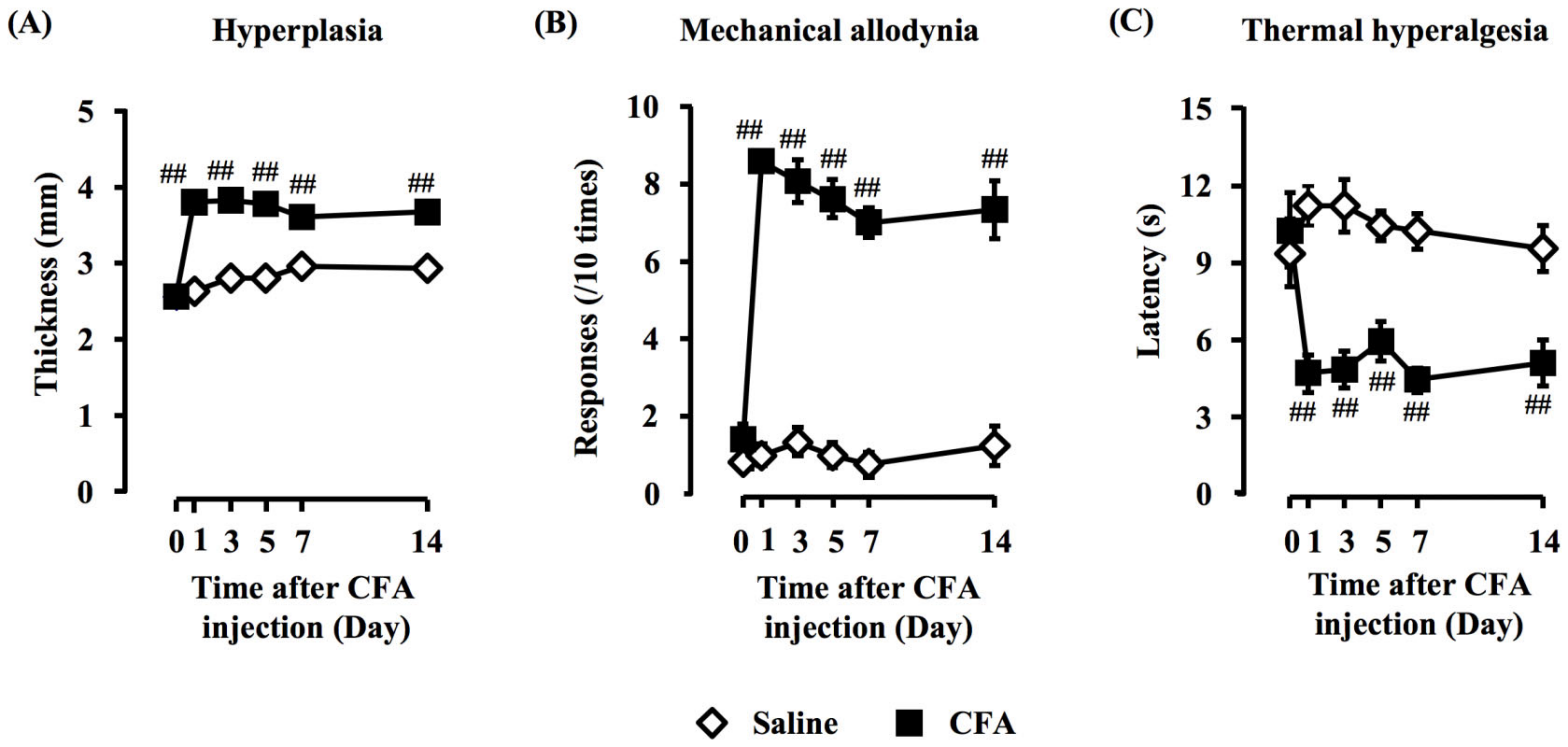
Development of hyperplasia, mechanical allodynia and thermal hyperalgesia after CFA injection. CFA (10 µL, 0.5 mg/ml) was injected into the plantar surface of a mouse hind paw. Intraplantar injection of CFA elicited persistent hyperplasia (A), mechanical allodynia (B) and thermal hyperalgesia (C) for day 14; data, mean ± S.E.M., Saline (n = 12), CFA (n = 12); ^##^ *p*<0.01, compared with saline (Student's *t*-test); ◊, saline; and ▪, CFA.

Long-lasting paw hyperplasia, persistent mechanical allodynia and thermal hyperalgesia (all *P*<0.01) were elicited in CFA-treated mice, compared with saline-injected control mice, appearing on day 1 and continuing until day 14. No pain behavior was observed in saline-injected mice (Fig. 1A–C).

### Changes in hypothalamic GPR40 expression in CFA-induced inflammatory chronic pain mouse model

**Figure 2:**
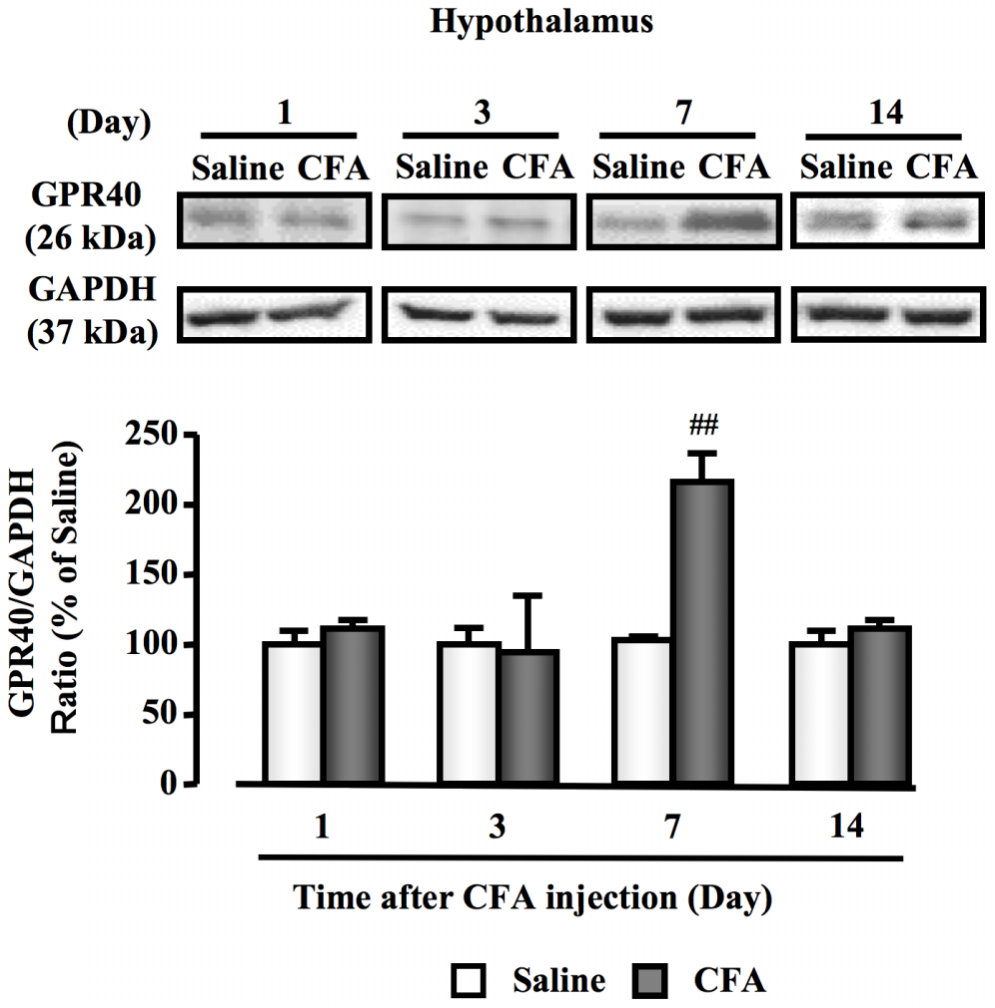
Changes in hypothalamic GPR40 expression in CFA-induced inflammatory chronic pain mouse model. White bar, saline-injection group; black bar, CFA-injection group; data, mean ± S.E.M.; Saline (n = 6), CFA (Day 1) (n = 6), CFA (Day 3) (n = 6), CFA (Day 7) (n = 6), CFA (Day 14) (n = 6); and ^##^ *p*<0.01, compared with saline (Student's *t*-test).

GPR40 protein expression in the hypothalamus was transiently and significantly increased at day 7 after CFA injection, in comparison with the saline group (*P*<0.05). However, there was no change in GPR40 expression in the hypothalamus at day 1, 3 or 14 after CFA injection, compared with saline groups (Fig. 2).

### Colocalization of GPR40 with neurons, but not astrocytes in the hypothalamus

**Figure 3:**
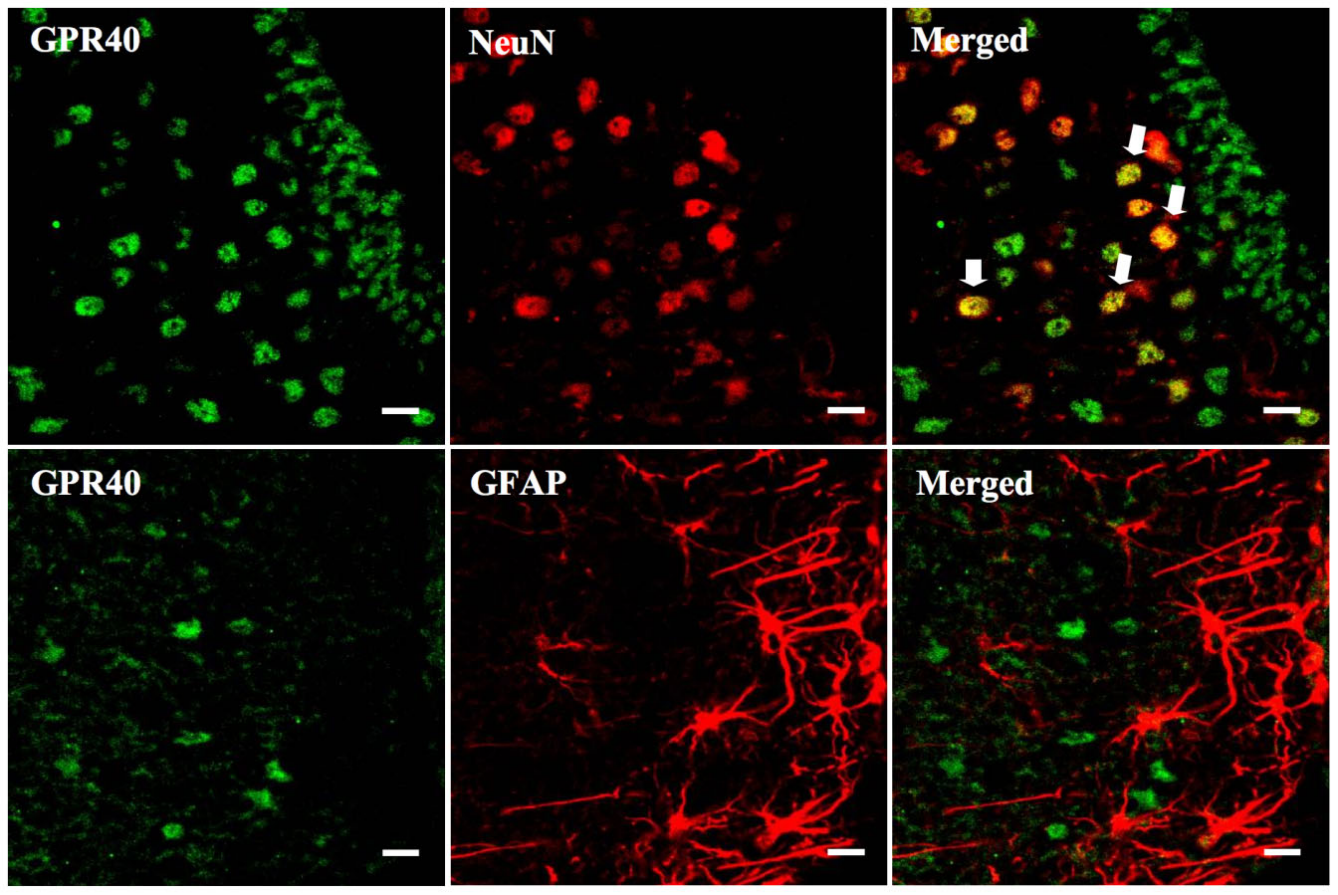
Colocalization of GPR40 with neurons, but not astrocytes in the hypothalamus. Localization of GPR40 with NeuN and GFAP in the hypothalamus of saline evaluated by double immunofluorescence staining; green, GPR40; red, NeuN (a neuron marker) and GFAP (an astrocyte marker). Scale bars: 10 µm.

GPR40-positive cells were observed in the hypothalamus of the saline group, with GPR40 colocalized with NeuN-positive cells, but not with GFAP (an astrocyte marker) in the saline group (Fig. 3).

### Time-dependent changes of astrocyte in the hypothalamus of CFA-induced inflammatory chronic pain mouse model

**Figure 4:**
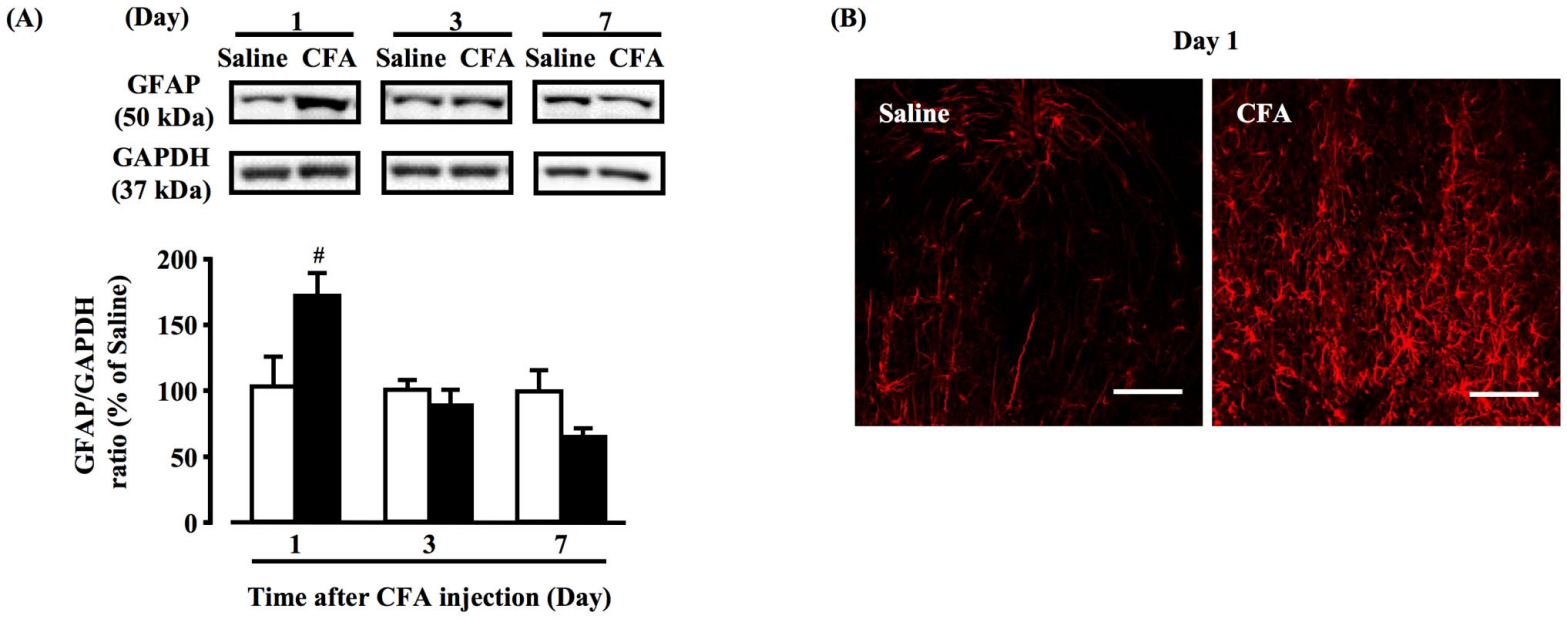
Time-dependent changes of astrocyte in the hypothalamus of CFA-induced inflammatory chronic pain mouse model. Representative Western blots of GPR40 and GAPDH levels in the hypothalamus after CFA injection are shown (A). White and black bars represent saline and CFA-injection groups, respectively. Immunofluorescence staining for GFAP in hypothalamus after saline or CFA injection (B). Scale bar, 50 µm; data, mean ± S.E.M; Saline (n = 6), CFA (Day 1) (n = 6), CFA (Day 1) flavopiridol 5 nmol (n = 6), CFA (Day 1)+flavopiridol 15 nmol (n = 6); ^#^ *p*<0.05, compared with saline (Student's *t*-test).

GFAP expression was markedly increased in the hypothalamus at day 1 after CFA injection, with no changes at day 3 or 7 after CFA injection, compared with the saline group (Fig. 4A, *P*<0.05). In addition, in immunohistochemical studies, increases in GFAP-positive cells were observed at day 1 after CFA injection (Fig. 4B).

### Effect of flavopiridol on CFA-elicited hyperplasia, GFAP increase, persistent mechanical allodynia and thermal hyperalgesia

**Figure 5:**
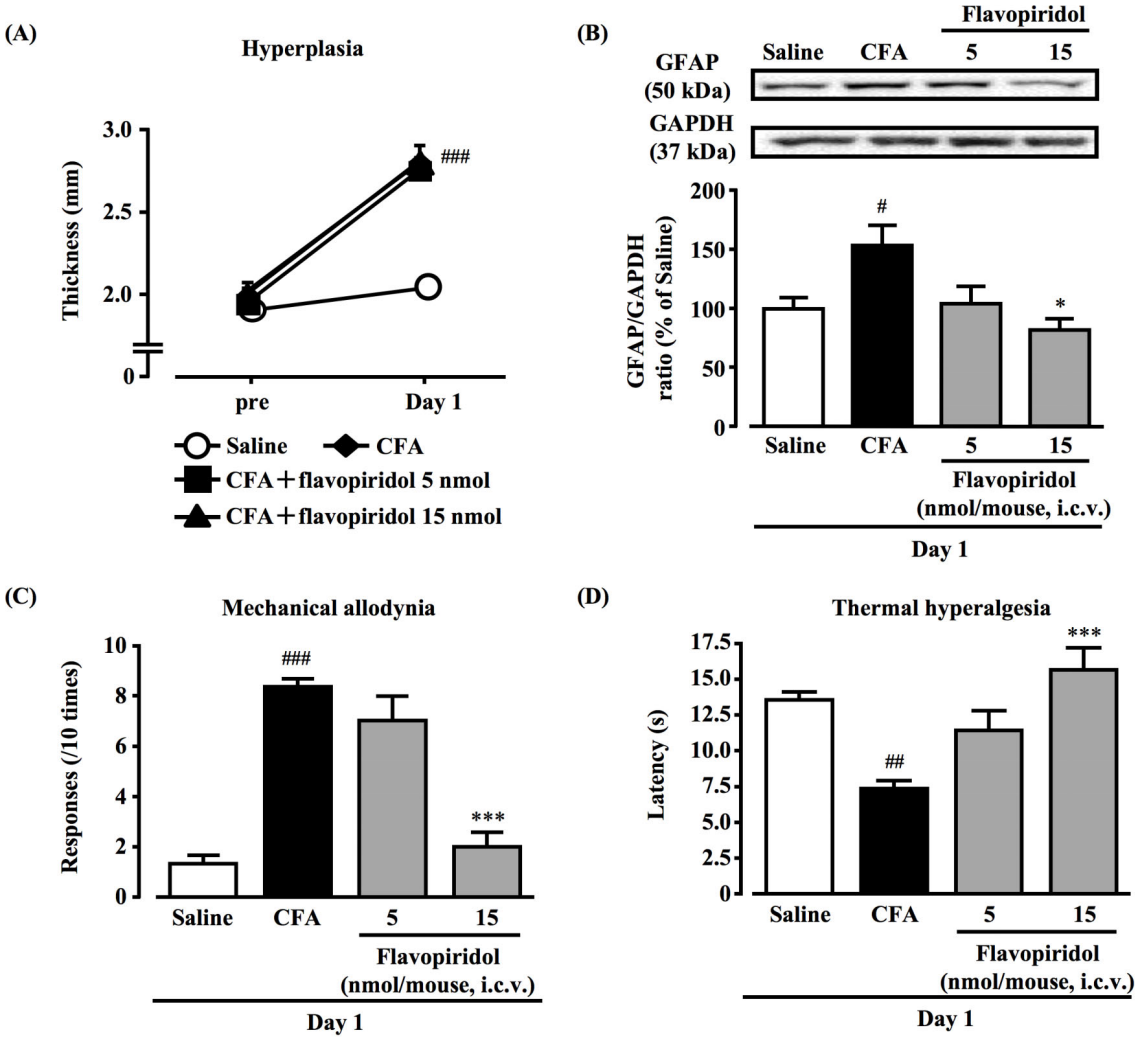
Effect of flavopiridol on CFA-elicited hyperplasia, GFAP increase, persistent mechanical allodynia and thermal hyperalgesia. Flavopiridol (5 and 15 nmol) was administered by i.c.v. injection into the left lateral ventricle of the mice twice a day (at 9:00 and 19:00) after CFA treatment. Hyperplasia of paw tissue was measured by means of a digital caliper (A). Representative Western blots of GFAP and GAPDH levels in the hypothalamus after CFA injection with or without flavopiridol are shown (B). Mechanical allodynia was evaluated using von Frey filaments (C). Thermal hyperalgesia of the hind paw was assessed using the plantar test (D); data, mean ± S.E.M.; Saline (n = 6), CFA (Day 7) (n = 6), CFA (Day 7)+flavopiridol 5 nmol (n = 6), CFA (Day 7)+flavopiridol 15 nmol (n = 6); ^#^ *p*<0.05, ^##^ *p*<0.01, ^###^ *p*<0.001, compared with saline; * *p*<0.05, *** *p*<0.001, compared with CFA (Scheffe's test).

At day 1 after CFA injection, hyperplasia had no effect in mice with the i.c.v. injection of flavopiridol (15 nmol, twice a day; Fig. 5A). The observed increase of GFAP protein expression in the hypothalamus at day 1 after CFA treatment was significantly suppressed by i.c.v. injection of flavopiridol (Fig. 5B, *P*<0.05). Behaviorally, CFA-treated mice with flavopiridol at day 1 showed significant recovery in mechanical allodynia and thermal hyperalgesia (Fig. 5C–D, all *P*<0.001).

Involvement of astrocytes in transient increases in hyperplasia, hypothalamic GPR40 protein expression increase, mechanical allodynia and thermal hyperalgesia in CFA-induced inflammatory chronic pain mouse model

**Figure 6:**
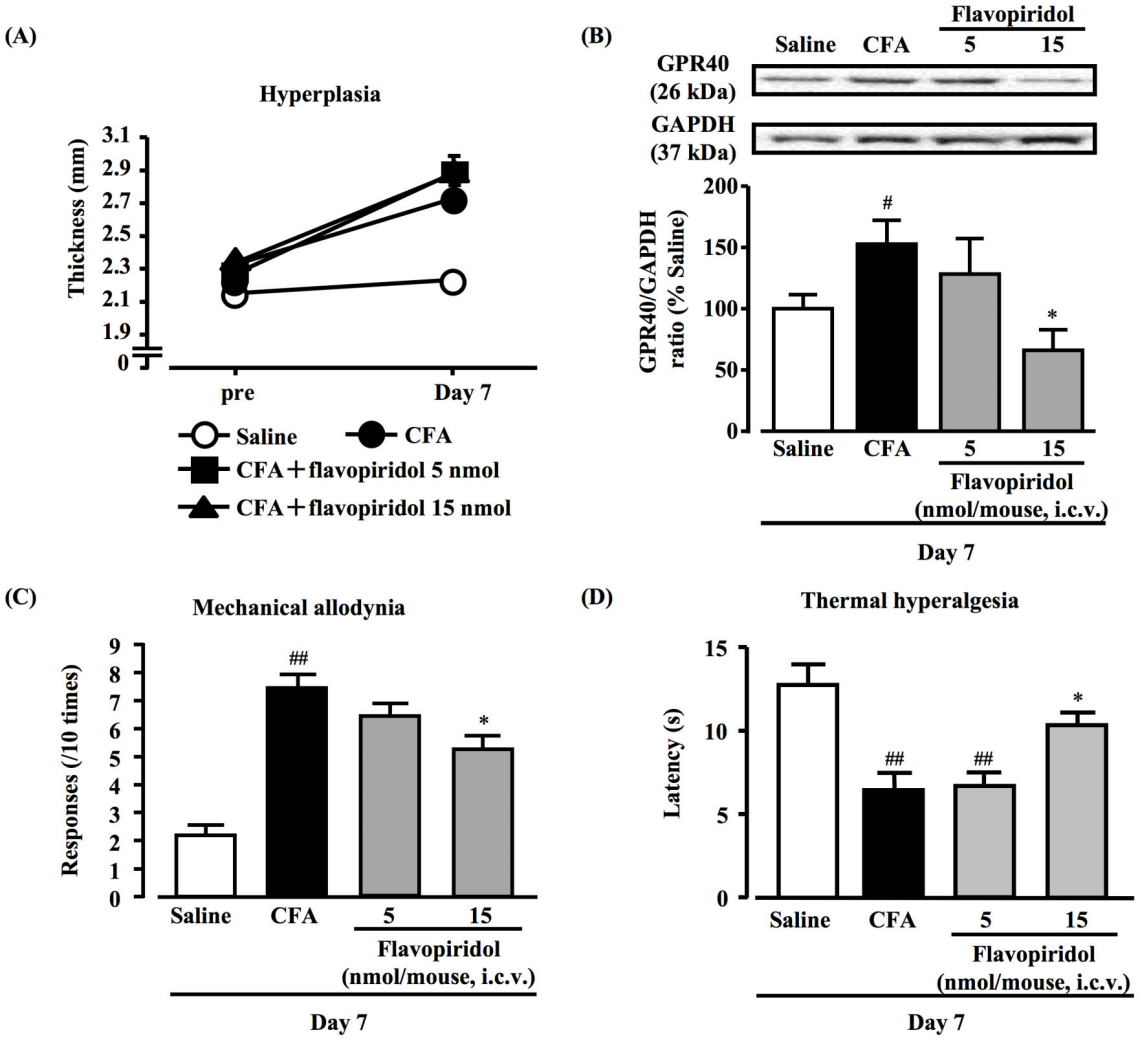
Effect of flavopiridol on CFA-elicited hyperplasia, GFAP increase, persistent mechanical allodynia and thermal hyperalgesia at day 7 after CFA injection. Flavopiridol (5 and 15 nmol) was administered by i.c.v. injection into the left lateral ventricle of the mice twice a day (at 9:00 and 19:00) after CFA treatment. Hyperplasia of paw tissue was measured by means of a digital caliper (A). Representative Western blots of GPR40 and GAPDH levels in the hypothalamus after CFA injection with or without flavopiridol are shown (B). White, black and grey bars represent saline, CFA and CFA+flavopiridol-injection groups, respectively. Mechanical allodynia was evaluated using von Frey filaments (C). Thermal hyperalgesia of the hind paw was assessed using the plantar test (D). Data, mean ± S.E.M.; Saline (n = 6), CFA (Day 7) (n = 6), CFA (Day 7)+flavopiridol 5 nmol (n = 6), CFA (Day 7)+flavopiridol 15 nmol (n = 6); ^#^ *p*<0.05, ^##^ *p*<0.01, compared with saline; *p<0.05, compared with CFA (Scheffe's test).

At day 7 after CFA injection, hyperplasia had no effect in mice with the i.c.v. injection of flavopiridol (Fig. 6A). GPR40 protein expression increases were significantly decreased to the level of the saline group in mice with the i.c.v. injection of flavopiridol (Fig. 6B, *P*<0.05). Concurrently, mechanical allodynia and thermal hyperalgesia at day 7 after CFA injection were also attenuated to the levels of the saline group (Fig. 6C–D, *P*<0.01 or *P*<0.05).

### FFAs compositions in the hypothalamus tissue

**Figure 7:**
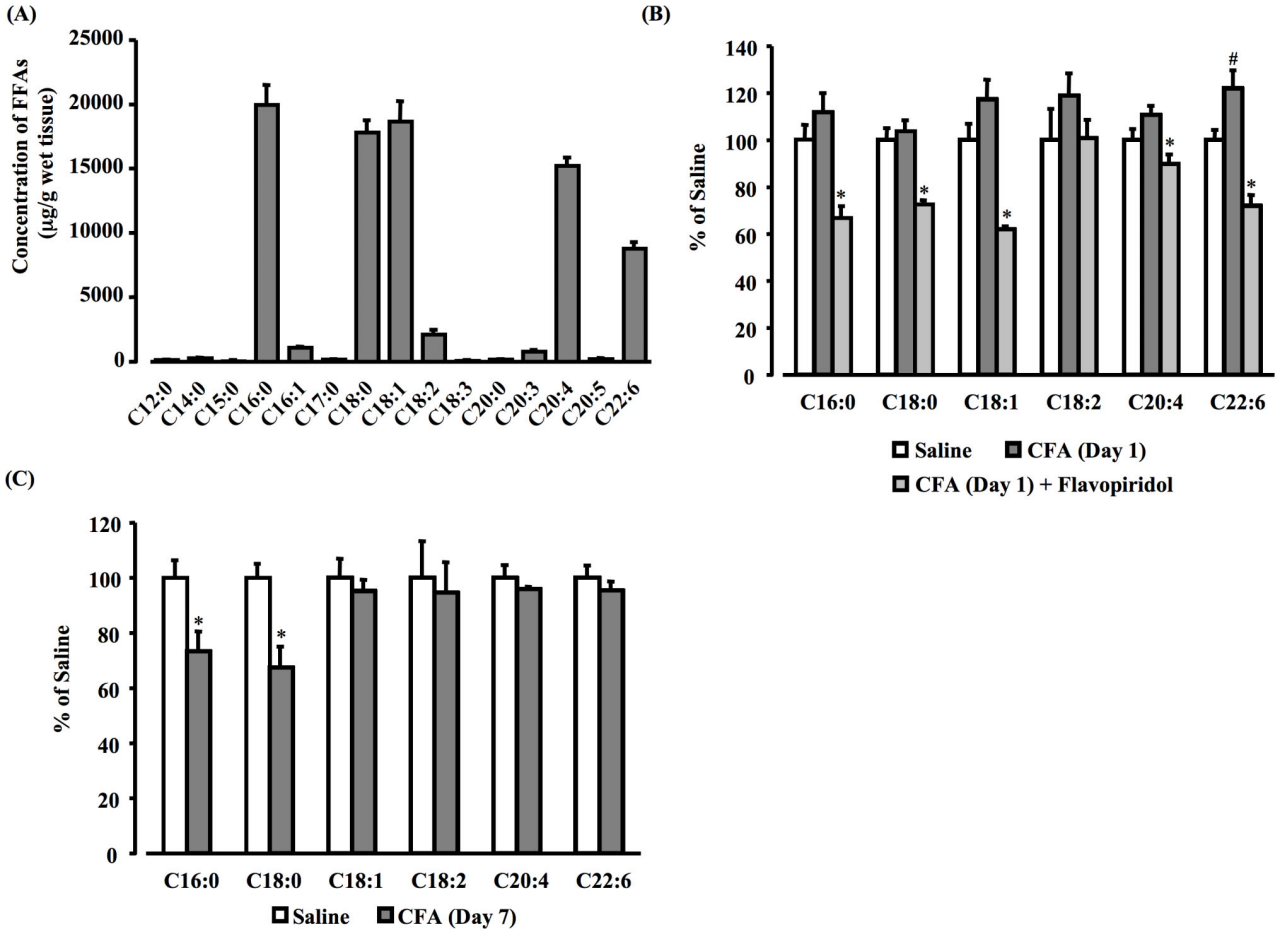
FFAs profile in the hypothalamus tissue. FFAs were analyzed with UHPLC-MS/MS using MRM; FFA profile in the hypothalamus of intact mice (A). The FFA composition ratio of palmitate (C16:0), stearate (C18:0), oleinic acid (C18:1), linoleic acid (C18:2), arachidonic acid (C20:4) and DHA (C22:6) at day 1 with or without flavopiridol injection (B) and day 7 after CFA injection (C). Data, mean ± S.E.M., Saline n = 4, CFA (Day 1) (n = 7), CFA (Day 1)+flavopiridol (15 nmol) (n = 4), CFA (Day 7) (n = 4); ^#^ *p*<0.05, compared with Saline (Scheffe's test), *p<0.05, compared with CFA (Scheffe's test).

All long chain FFAs were detected in the hypothalamus of normal mice. Notably, saturated palmitate (C16:0) and stearate (C18:0), monosaturated oleinic acid (C18:1), polyunsaturated linoleic acid (C18:2), arachidonic acid (C20:4) and DHA (C22:6) were abundantly expressed in the hypothalamus, and the FFA contents (µg/g wet tissue) were 31965±3977, 24516±2408, 29711±4011, 2907±759, 18419±1654 and 13686±1154, respectively (Fig. 7A). The ratio of free DHA (C22:6) was significantly increased at day 1 after CFA injection compared with the saline-treated control group, and another type of FFA in the hypothalamus tissue tended to increase compared with the control group injected with saline (Fig. 7B). This increase was inhibited by the i.c.v. pretreatment of flavopiridol. On the other hand, at day 7 after CFA treatment, the levels of free palmitate (C16:0) and stearate (C18:0) were significantly decreased compared with the control group, and other fatty acids including DHA returned to the control levels compared with day 1 after CFA injection (Fig. 7C).

### Antinociceptive effect of GW9508 or DHA on mechanical allodynia and thermal hyperalgesia of CFA-treated mice

**Figure 8:**
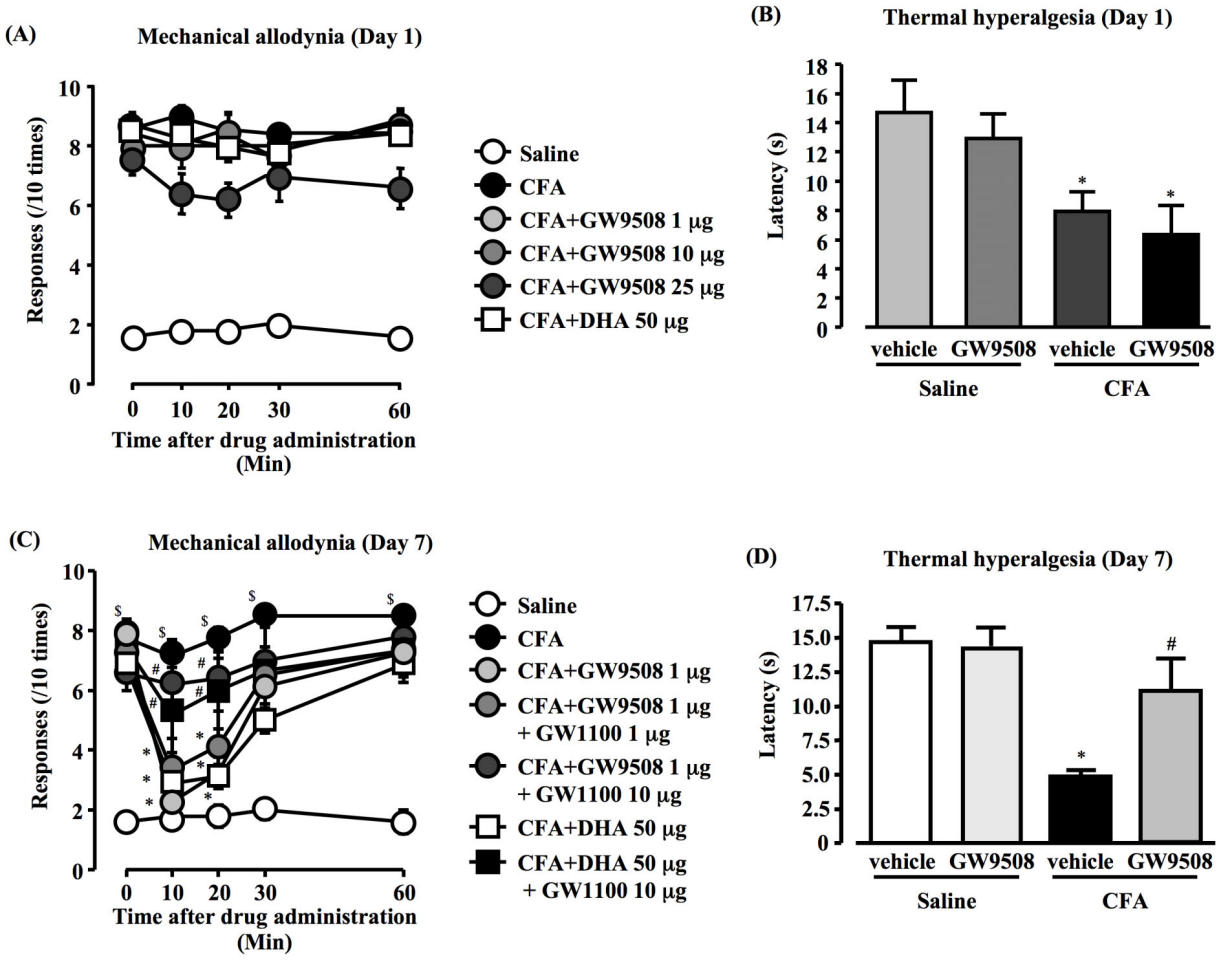
Antinociceptive effect of GW9508 and DHA on CFA-induced mechanical allodynia and thermal hyperalgesia. GW9508 (1.0 µg) and DHA (50 µg) i.c.v. administered in CFA-injected mice, whereas GW1100 (10 µg) was administered via the i.c.v. route 10 min before GW9508 or DHA injection; CFA-induced mechanical allodynia and thermal hyperalgesia are examined at day 1 (A, B) and 7 (C, D) post-injection; data, mean ± S.E.M.; Saline (n = 6), CFA (Day 1) (n = 6), CFA (Day 1)+GW9508 (1 µg) (n = 6), CFA (Day 1)+GW9508 (10 µg) (n = 6), CFA (Day 1)+GW9508 (25 µg) (n = 6), CFA (Day 1)+GW9508 (25 µg) (n = 6), CFA (Day7) (n = 6), CFA (Day 7)+GW9508 (1 µg) +GW1100 (1 µg) (n = 6), CFA (Day 7)+GW9508 (1 µg) +GW1100 (10 µg) (n = 6), CFA (Day 7)+DHA (50 µg) (n = 6), CFA (Day 7)+DHA (50 µg)+ GW1100 (10 µg) (n = 6); ^$^ *p*<0.05, compared with saline; *p<0.05, compared with CFA; ^#^ *p*<0.05, compared with CFA+GW9508 (Scheffe's test).

At day 1 after CFA injection, mechanical allodynia (Fig. 8A) and thermal hyperalgesia (Fig. 8B) was not affected by the i.c.v. treatment of DHA (50 µg) or GW9508 (1.0 µg).

On the other hand, administration of DHA (50 µg) or GW9508 (1.0 µg) significantly suppressed mechanical allodynia (Fig. 8C) and thermal hyperalgesia (Fig. 8D) at day 7 after CFA injection. Furthermore, this effect peaked at 10 min, and lasted for at least 20 min. These effects were inhibited by pretreatment with GW1100 (10 µg, Fig. 8C, D, *P*<0.05). When 1% DMSO was given as a control or GW1100 was used alone, there was no effect on CFA-induced inflammatory pain.

### Colocalization of GPR40 with POMC neuron

**Figure 9:**
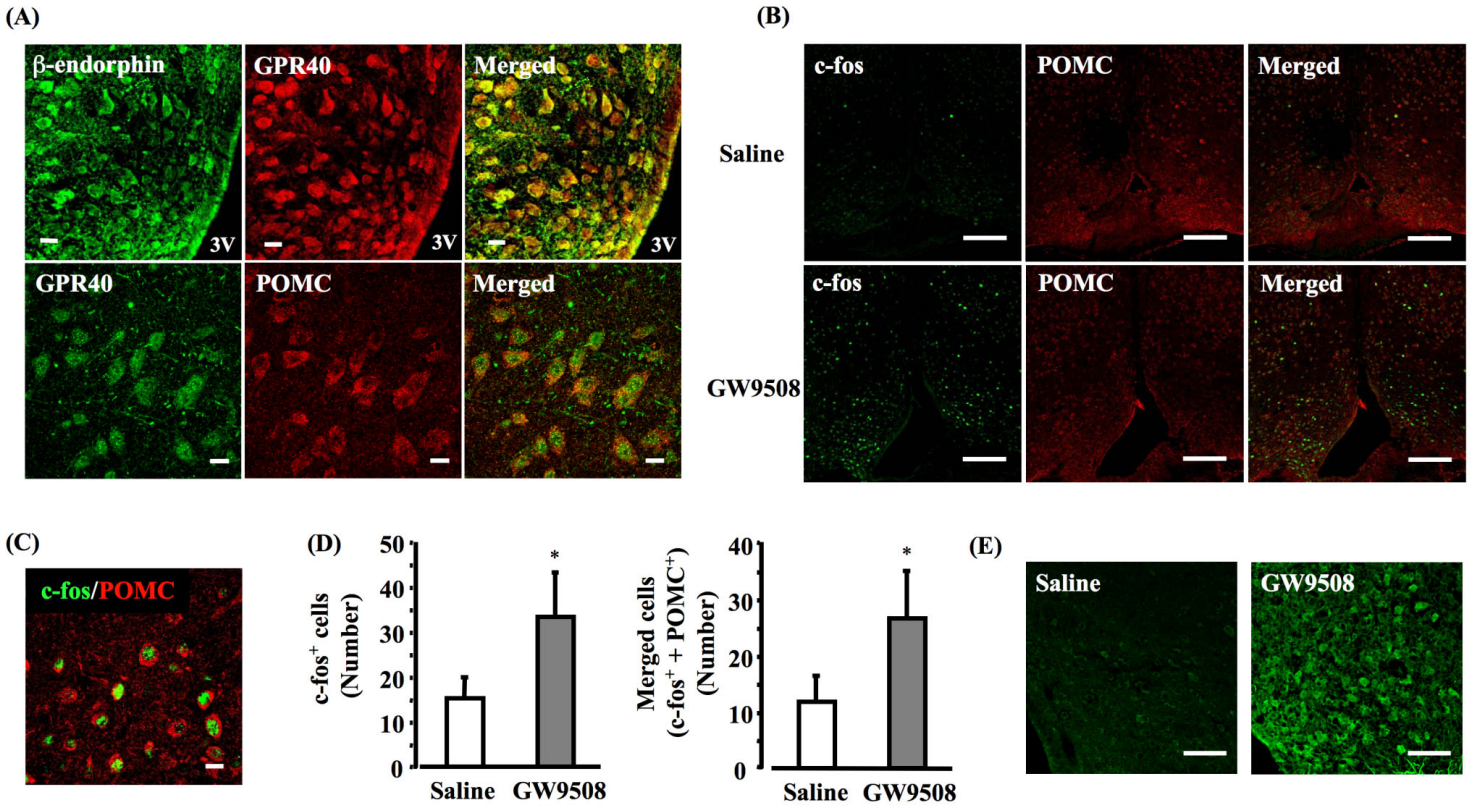
GW9508 injection activates POMC neuron, and releases β-endorphin in the arcuate neucrei of hypothalamus. Colocalization of GPR40 with POMC (a proopiomelanocortin neuron marker) and β-endorphin (A). Colocalization in the hypothalamus was evaluated with double immunofluorescence staining (green: GPR40; red: POMC; or red: GPR40, green β-endorphin). Scale bars: 10 µm. GW9508 administration causes induction of c-Fos protein in the arcuate nucleus. Confocal microscopic images of neurons double-labeled with POMC and c-Fos in the arcuate nucleus of the hypothalamus following the administration of GW9508 (1 µg/mouse, i.c.v.) (B). The image shows high magnifications of colocalization of neurons double-labeled with POMC and c-Fos in the arcuate nucleus of the hypothalamus (C) (green: c-Fos; red: POMC). Scale bars: 10 µm. Data summary is shown on the bottom, data, mean ± S.E.M.; Saline (n = 5), GW9508 (n = 5); *p<0.05, compared with Saline (D). Immunofluorescence staining for β-endorphin in the hypothalamus following the administration of GW9508. Scale bars: 50 µm (E).

GPR40 protein colocalized with β-endorphin and POMC in the hypothalamus (Fig. 9A). A single i.c.v. injection of GW9508 (1.0 µg) significantly increased the number of c-Fos-positive cells in the arcuate nucleus of the hypothalamus, compared with the saline-treated group. Furthermore, there was an increase in the number of neurons double-stained for c-Fos and POMC (*P*<0.05) in the mice treated with GW9508, compared with the saline-treated group. In contrast, in saline-injected mice, there were almost no c-Fos or POMC-positive neurons (Fig. 9B–D). The i.c.v. injection of GW9508 increased the positive cells of β-endorphin in the arcuate nucleus of the hypothalamus compared with the saline-treated control group (Fig. 9E).

## Discussion

Growing evidence has indicated that *n*-3 PUFAs have beneficial effects on human health [27], [28], [29], [30]. However, the mechanism of *n*-3 PUFA-modulated signaling remains unknown. Here, we use an inflammatory chronic pain mouse model to present the first evidence that hypothalamic GPR40 may contribute to the pain control system.

At day 7 after CFA injection, an increase of GPR40 expression was observed in the hypothalamus, which is the region associated with production of β-endorphin and with the pain control system. Although the GPR40 protein levels in the brain were initially expected to change in the early stages of CFA-induced pain, such changes did not occur immediately after CFA injection and instead resulted in increases by day 7 after CFA injection. This suggests that GPR40 protein expression may be affected by continuous pain stimuli. On the other hand, FFAs including DHA significantly increased in the hypothalamus at 1 day, but not day 7, after CFA injection, suggesting that this increase of DHA may contribute to the activation of GPR40 signaling and to suppressing pain signals in the brain although GPR40 protein had no change against CFA-induced pain. Furthermore, our results indicate that an increase of FFAs may initiate suppression of CFA-induced pain at an early stage. Therefore, it is suggested here that long-chain FFAs including DHA mainly act on GPR40 in the brain, and that the activation of this receptor suppresses the pain signal. Previous reports have shown that *n*-3 PUFAs have beneficial effects on acute or chronic inflammatory diseases [31]. Furthermore, Arita et al. have shown that *n*-3 PUFAs are enzymatically converted to bioactive metabolites during acute inflammation and resolution [3]. This mechanism is involved in inflammatory response regulation by local production of *n*-3 PUFA-derived lipid mediators such as resolvin and neuroprotectin [3], [32]. It has been reported that DHA decreases with age in brain neurons [33] and with psychiatric disorders and/or neurodegenerative diseases such as Alzheimer's disease, schizophrenia and mood disorders [34]. Thus, it is thought that *n*-3 PUFAs have a critical role in both physiological and pathologic responses. In this study, at day 7 after CFA injection, the FFA levels were not changed and/or decreased compared with the control group. It is thought that hypothalamic FFAs were continuously released by pain stimuli, and may cause dysfunction of the GPR40-mediated pain control system via decreasing FFAs 7 days after CFA injection. Considering these reports and the present results, increased GPR40 expression may be a compensatory reaction caused by the decreased release of FFAs. These FFAs in the hypothalamus may continue to suppress activation of pain signaling.

Another important finding from the present work is that GPR40-induced antinociception might be regulated by astrocytes. Glial cells, consisting of microglia and astrocytes, constitute more than 70% of the total cell population in the central nervous system [35], [36]. Of these cells, astrocytes have intimate contact with synaptic elements and are thus likely to serve as key links between a peripheral disease process and detrimental brain responses. Interestingly, astrocytes cooperate in the local synthesis and release of *n*-3 PUFAs, collectively maintaining a brain environment enriched in *n*-3 PUFAs [37]. Furthermore, DHA is readily released from astroglial membranes under basal and stimulated conditions and supplied to the neurons [37], [38], [39], [40]. In the present study, we found a significant increase of both GFAP protein expression and FFAs levels at day 1 after CFA injection. Consequently, in this model the increase of GFAP protein expression may affect the ratio of changes in FFA levels in the hypothalamus 1 day after CFA injection. Remarkably, double immunofluorescence techniques revealed here that GPR40 was co-localized on neurons, which is supported by a previous report showing that GPR40 exists on primate neurons [41]. From these results, we hypothesize that GPR40 expressed on neurons may be regulated by astrocytes accompanying the variation of FFA release. That is, astrocyte proliferation accompanying the increase of FFA release early after CFA injection may help increase GPR40 expression in the state of chronic pain. To test this hypothesis, a further examination was conducted using the cell inhibitor flavopiridol, which inhibits astrocyte proliferation *in vitro* and *in vivo* [42]. Flavopiridol inhibits cyclin-dependent kinases, leading to reduced cyclin D1 expression and cell arrest in G1 or at the G2/M transition [43]. Furthermore, cyclin D1 is essential to astrocyte proliferation [44], and flavopiridol treatment suppresses neuropathic pain, mediated through inhibition of astrocyte proliferation [45]. In the present study, the i.c.v. injection of flavopiridol attenuated both mechanical allodynia and thermal hyperalgesia at day 7 after CFA injection. However, this effect was weak compared with the result for day 1 after CFA. These phenomena may be caused by other mechanisms such as activation of the immune system including macrophages, neutrophils and granulocytes via tissue injury with CFA-induced inflammatory chronic pain and plastic change of neurons. Therefore, we conclude that activation of hypothalamic astrocytes may contribute to regulation of pain in an early phase, and the ratio of changes of FFA levels might contribute to modulation of GPR40 expression. However, flavopiridol has an effect on myeloid cells and neutrophils apart from being an inhibitor to cell cycling [46], [47]. Thus, we cannot exclude the possibility that the effects on both mechanical allodynia and thermal hyperalgesia are not mediated by cytokines from leukocytes or by direct action of flavopiridol on neurons.

To clarify the mechanisms underlying antinociceptive action mediated through GPR40, the effects of GW9508 and DHA on mechanical allodynia and thermal hyperalgesia were examined in a CFA-induced pain mouse model. Interestingly, i.c.v. injection of GW9508 and DHA suppressed both mechanical allodynia and thermal hyperalgesia at day 7, but not 1 day, after CFA injection. These effects are likely related to the GPR40 protein levels because GPR40 expression was increased at day 7 after CFA injection. FFAs are probably not enough around GPR40 to induce antinociception at day 7 after CFA injection. In fact, in this condition, almost all FFAs were decreased compared with their levels at day 1 after CFA injection. That is, we believe that a gradual loss of FFAs will occur in a late phase of CFA-induced pain, while the mechanism of a post-GPR40 mediated system will be activated by an increase of GPR40 protein levels. In fact, i.c.v. injection of GW9508 produced antinociceptive effects on both mechanical allodynia and thermal hyperalgesia at day 7 after CFA injection. On the other hand, this agonist did not affect the pain reactivity at 1 day after CFA injection. Under these conditions, FFAs will be released strongly more than in the normal state to decrease pain signals in the brain, depending on whether astrocytes are activated by CFA injection. Thus, this result indicates that an excess of FFAs released from astrocytes may be acutely caused by hyposensitivity of GPR40 signaling, and therefore i.c.v. injection of GPR40 agonist may not suppress CFA-induced mechanical allodynia and thermal hyperalgesia.

Furthermore, we performed double immunofluorescence to identify the neuronal cell types expressing GPR40. Interestingly, GPR40 colocalized with POMC in the arcuate nucleus of the hypothalamus. POMC is a precursor of several active peptides, including adrenocorticotropin, β-endorphin and melanotropin (α, β and γ-MSH) [48]. POMC neurons, which project from the hypothalamic arcuate nucleus to the periaqueductal gray matter (PAG), play major roles in the development of antinociception to noxious stimuli, non-noxious stress and nociceptive stimuli associated with inflammation [49], [50], [51]. In particular, β-endorphin, a part of the endogenous opioid system, plays an important role in the descending pain inhibitory system in the supraspinal areas [52]. In fact, β-endorphin is released into the plasma following exposure to a painful stimulus [53], [54], [55]. GPR40 protein expression colocalized with β-endorphin in the hypothalamus area, suggesting that hypothalamic GPR40 may be expressed on POMC neurons, which are associated with production of β-endorphin. In our previous study, the i.c.v. injection of DHA and GW9508, a GPR40 agonist, had antinociceptive effects through the increase of β-endorphin in the arcuate nucleus of the hypothalamus, suggesting the GPR40 signaling may relate to the production of β-endorphin. Furthermore, the i.c.v. administration of a GPR40 agonist, GW9508, significantly increased the number of c-Fos-positive cells in the arcuate nucleus of the hypothalamus compared with saline-treated group. In this study, the number of POMC-positive cells also markedly increased compared with the saline group, and there was an increase in the number of neurons double-labeled for c-Fos and POMC. Taken together, we concluded that the GPR40 mediated signaling system might produce antinociceptive effects through the release of β-endorphin.

In conclusion, the important functional role of fatty acids, their receptors, and their metabolites in both the onset and suppression of pain has become increasingly apparent in recent years. The present findings support the idea that GPR40 signaling in the supraspinal area may contribute to regulation of the pain control system. In addition, GPR40 expressed on POMC neurons was shown to possibly control excitation signaling caused by inflammatory chronic pain. Taken together, application and study of a GPR40 agonist in this model might provide valuable information regarding a novel therapeutic approach for pain control in the future.
